# Structure of the Reductase Domain of a Fungal Carboxylic Acid Reductase and Its Substrate Scope in Thioester and Aldehyde Reduction

**DOI:** 10.1021/acscatal.2c04426

**Published:** 2022-12-06

**Authors:** Bastian Daniel, Chiam Hashem, Marlene Leithold, Theo Sagmeister, Adrian Tripp, Holly Stolterfoht-Stock, Julia Messenlehner, Ronan Keegan, Christoph K. Winkler, Jonathan Guyang Ling, Sabry H.H. Younes, Gustav Oberdorfer, Farah Diba Abu Bakar, Karl Gruber, Tea Pavkov-Keller, Margit Winkler

**Affiliations:** † acib - Austrian Center of Industrial Biotechnology, Krenngasse 37, 8010Graz, Austria; ‡ Institute of Molecular Biosciences, University of Graz, Humboldtstraße 50, 8010Graz, Austria; § Institute of Molecular Biotechnology, Graz University of Technology, Petersgasse 14, 8010Graz, Austria; ⊥ Department of Biological Sciences and Biotechnology, Universiti Kebangsaan Malaysia, 43600Bangi, SelangorMalaysia; ∥ BioHealth Field of Excellence, University of Graz, 8010Graz, Austria; ¶ BioTechMed-Graz, 8010Graz, Austria; # Institute for Biochemistry, Graz University of Technology, Petersgasse 12, 8010Graz, Austria; & Rutherford Appleton Laboratory, Research Complex at Harwell, UKRI-STFC, DidcotOX11 0FA, United Kingdom; ◆ Institute of Chemistry, University of Graz, Heinrichstraße 28, 8010Graz, Austria; ▼ Department of Chemistry, Faculty of Science, Sohag University, Sohag82524, Egypt; ○ Department of Biotechnology, TU Delft, Van der Maasweg 9, 2629HZDelft, The Netherlands

**Keywords:** carboxylic acid reductase, reductase domain, X-ray crystallography, short-chain dehydrogenase/reductase, thioester

## Abstract

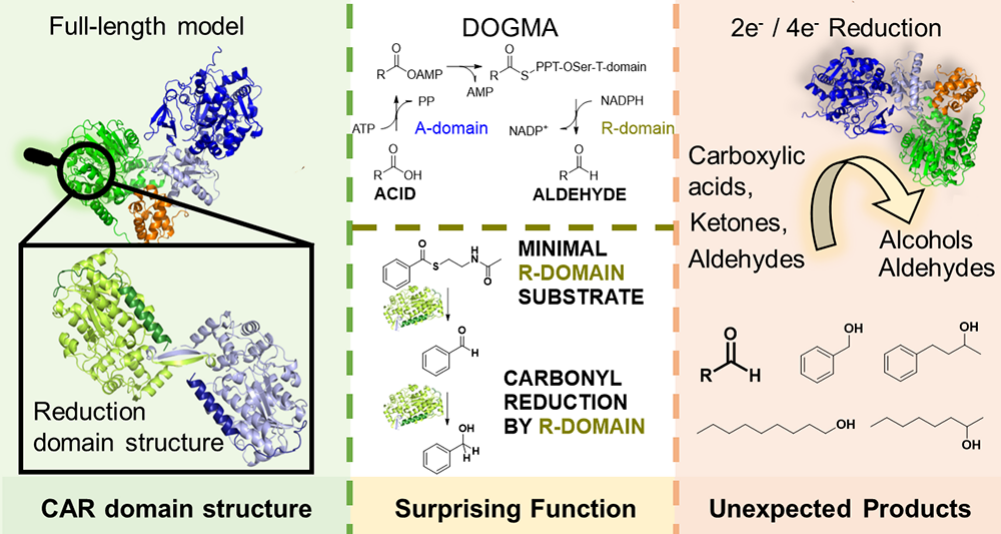

The synthesis of aldehydes from carboxylic acids has long been a challenge in chemistry. In contrast to the harsh chemically driven reduction, enzymes such as carboxylic acid reductases (CARs) are considered appealing biocatalysts for aldehyde production. Although structures of single- and didomains of microbial CARs have been reported, to date no full-length protein structure has been elucidated. In this study, we aimed to obtain structural and functional information regarding the reductase (R) domain of a CAR from the fungus *Neurospora crassa* (*Nc*). The *Nc*CAR R-domain revealed activity for *N*-acetylcysteamine thioester (S-(2-acetamidoethyl) benzothioate), which mimics the phosphopantetheinylacyl-intermediate and can be anticipated as the minimal substrate for thioester reduction by CARs. The determined crystal structure of the *Nc*CAR R-domain reveals a tunnel that putatively harbors the phosphopantetheinylacyl-intermediate, which is in good agreement with docking experiments performed with the minimal substrate. *In vitro* studies were performed with this highly purified R-domain and NADPH, demonstrating carbonyl reduction activity. The R-domain was able to accept not only a simple aromatic ketone but also benzaldehyde and octanal, which are typically considered to be the final product of carboxylic acid reduction by CAR. Also, the full-length *Nc*CAR reduced aldehydes to primary alcohols. In conclusion, aldehyde overreduction can no longer be attributed exclusively to the host background.

Carboxylic acid reductases (CARs) evolved to produce aldehydes from carboxylates. The aldehyde functionality is a valuable chemical moiety to make a variety of products, and CARs are becoming standard tools for this transformation.^1^

On the molecular level, CARs catalyze an ATP- and NADPH-dependent reaction cascade (Scheme 1A).^2,3^ CARs consist of functional domains that are each responsible for a respective step, namely the adenylation of the acid (A-domain), its transthiolation using a phosphopantetheine moiety (T-domain or peptidyl carrier protein (PCP)-domain), and finally the reduction of the enzyme-bound thioester intermediate to the aldehyde in the reductase domain (R-domain). The substrate is shuttled as covalent thioester **1** from domain to domain through large-scale domain movements.^4^ This intrinsic flexibility of full-length CARs makes them challenging targets for structural characterization. However, some mechanistic and structural insight was deduced from crystallized single and didomains of bacterial CARs.^4^ CARs can be divided into five clans: The group of bacterial CARs belongs to type I, while type III consists of CARs from Ascomycetes, type IV of CARs from Basidiomycetes and type II represents a mixed group of fungal origin.^5^ The fifth type of enzymes of similar domain architecture is specialized in reducing aromatic amino acids.^6^ Sequence similarity between different CAR types is low,^7^ hence the structural insight from one clan is of limited value for the others. In particular, the selectivity of the R-domain for two-electron reduction (Scheme 1B) requires more attention,^4^ especially as hypothesized important residues in bacterial CARs are not conserved in other CAR types.

**Scheme 1:**
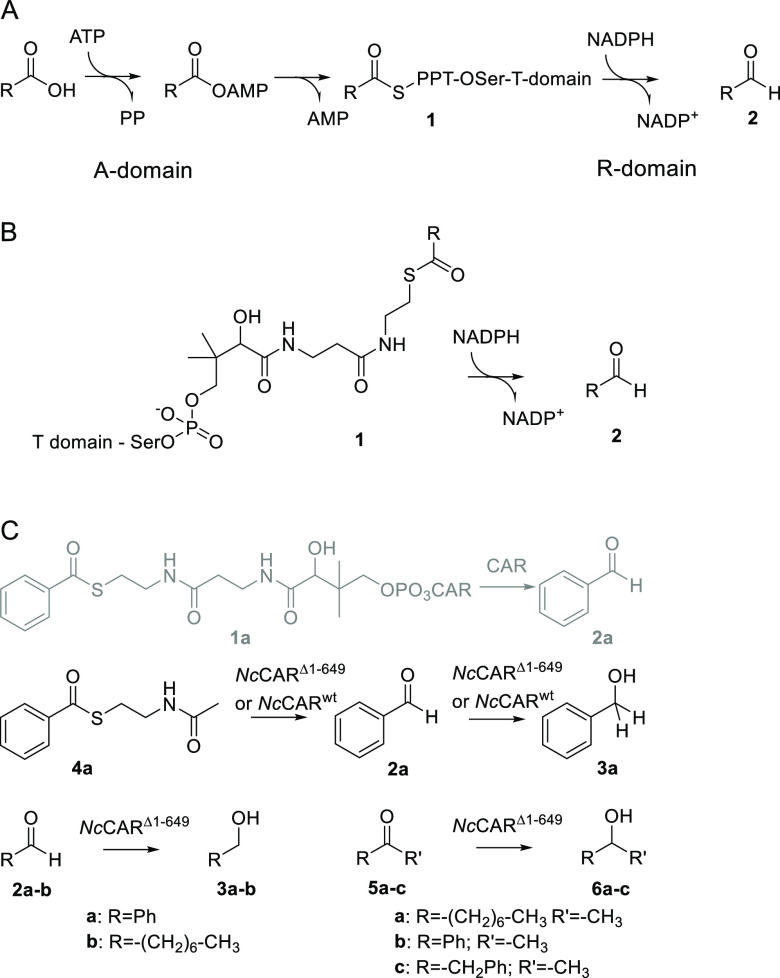
(A) Overall Reaction Cascade from Acid to Aldehyde Catalyzed by CARs; (B) General Two-Electron Reduction in the R-Domain (C) Reduction of Phosphopantetheine-Tethered Benzoate in CARs (Cofactors Omitted) in Grey and Extended Reaction Scope of the Short Chain Alcohol Dehydrogenases-Like R-Domain of *Nc*CAR in Black

We devised an approach for crystallizing single domains of a fungal CAR (type III). Crystals of the *Neurospora crassa* (*Nc*) CAR^8^ reduction domain were first derived from proteolytic cleavage of heterologously expressed full-length *Nc*CAR equipped with WELQut cleavage sites at domain boundaries. Subsequently, the R-domain (*Nc*CAR^Δ1–649^) was produced via a truncated open reading frame to increase protein yield. Based on the crystal structure of *Nc*CAR^Δ1–649^, activity assays with alternative substrates and docking experiments, we postulate how the enzyme-thioester intermediate is reduced. We identified the crucial part of the phosphopantetheinyl arm that leads to the productive mode. Free thioesters are not accepted as substrates by the enzyme, due to the lack of *N*-acylcysteamine.

## Structure of the *Nc*CAR R-Domain

**Figure 1:**
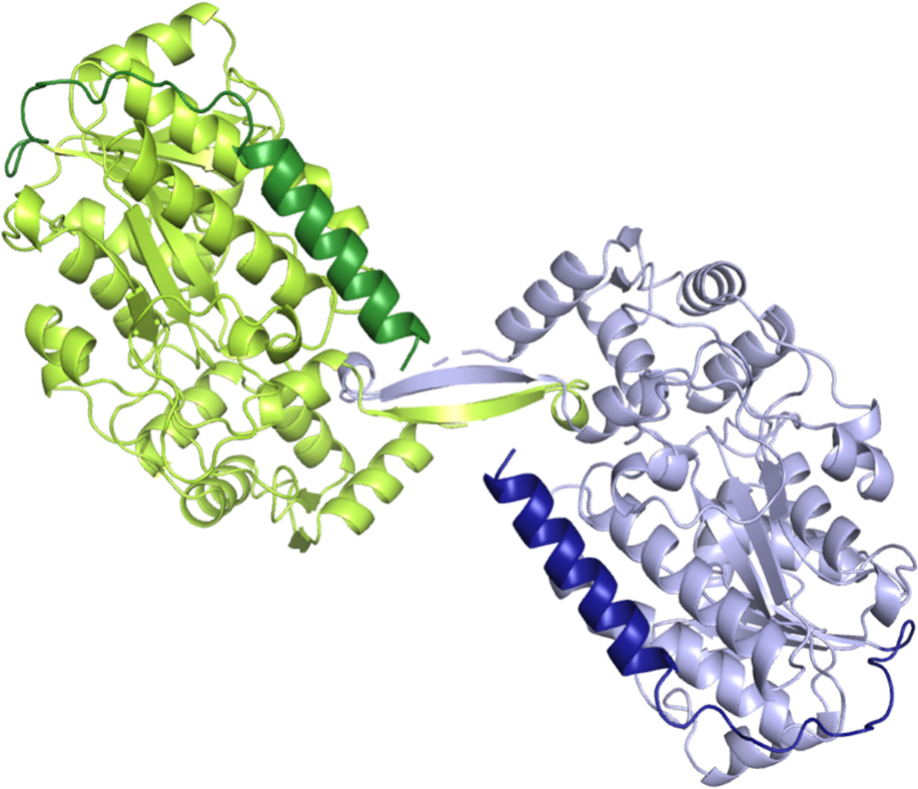
Overall structure of the *Nc*CAR R-domain dimer. Individual monomers are shown in cartoon representation. Chain A is shown in light green with the respective linker in dark green and Chain B is shown in light blue and dark blue, respectively.

Bacterial CARs have been demonstrated to be highly dynamic enzymes employing excessive domain movements to achieve a productive interplay of different domains.^4^ To understand the mechanism underlying the strict two-electron reduction of thioesters and the substrate specificity of fungal CARs we have solved the crystal structure of the *Nc*CAR reductase domain^9−12^ (PDB-code 8AEP). The respective construct that was crystallized in this work consists of the R-domain subunit (residues 684–1052) and a linker (residues 649–683) that connects the R-domain to the PCP-domain (SI, Table S1). The construct will further be referred to as R-domain in this article. It was found to adopt the Rossmann-fold with an NADPH binding site. It crystallized as a dimer, with a joint beta-sheet between the two monomers (Figure 1).

The interface between R-domain monomers was analyzed using the PISA web server to estimate the biological relevance. The interface formed by residues Ala981-Ser1008 shows a calculated area of 1213 Å^2^. The low Complex Formation Significance Score (CSS) of 0.178 indicates no biological relevance of this dimerization event. The residues Asp1002-Thr1006 form an antiparallel beta-sheet with their respective counterparts (Figure 1 and SI, Figure S3). In particular, the beta-sheet formed between the two domains aroused our interest, as the separate beta-strands are not expected to be stable without their respective counterpart. Considering that the R-domain represents a subdomain of the full-length *Nc*CAR, the respective residues might be a part of a dimerization area collectively formed by the A-, PCP- and R-domain. Nevertheless, full-length *Nc*CAR was described as a monomeric enzyme,^13^ and this was confirmed via size exclusion chromatography (Figure S5). Next to the predominant monomeric form, also higher oligomeric states can be observed and their relevance will need to be elucidated in future experiments (Figure S5).

To further evaluate the oligomeric state of the protein in solution, SAXS-measurements were performed. The best fit of the experimental data is depicted in Figure S1 and indicates the presence of 69% dimer and 31% monomer in solution (SI, Figure S1). The exact nature of the biological relevant oligomerization state will have to be elucidated with the full-length *Nc*CAR.

**Figure 2:**
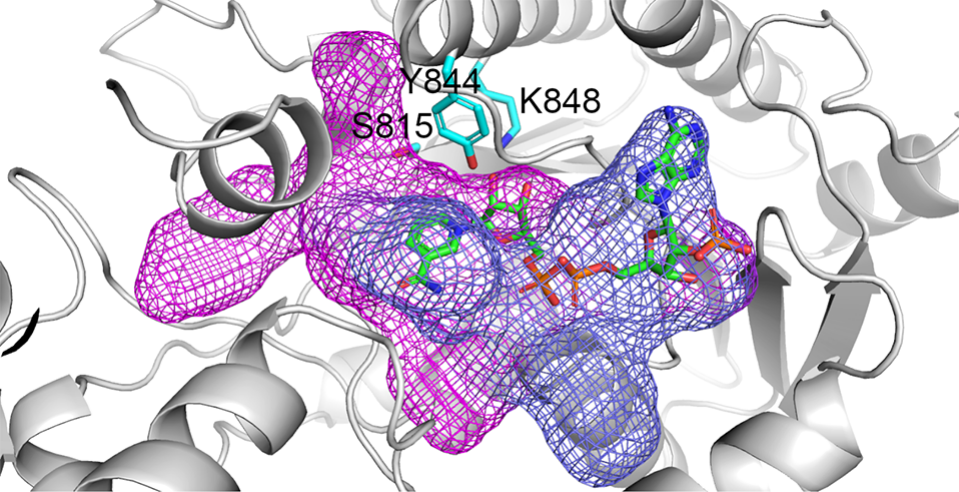
Active site of the R-domain of *Nc*CAR, visualized in PYMOL using the CavMan plugin. The cavity is depicted as mesh, while the NADPH cofactor is depicted as sticks. Highly accessible parts of the cavity are depicted in blue, areas with restricted accessibility in pink.

The closest structural homologue of the *Nc*CAR R-domain (24% sequence identity) is the R-domain from *Segniliparus rugosus*, *Sr*CAR (PDB-code 5MSP).^4^ Both enzymes were found to be NADPH-dependent, and the key residues for cofactor binding are highly conserved. Therefore, the respective NADPH binding mode in *Nc*CAR was modeled as observed in *Sr*CAR and visualized (Figure 2).

The *Nc*CAR cavity is broad and open to the solvent on one side, narrowing down to a tunnel that leads to the opposite side of the protein. There, the C-terminal end of an α helix (Trp954 to Tyr964) forms a putative sulfate or phosphate binding site (SI, Figure S2). In contrast to the tunnel we observed for *Nc*CAR, the *Sr*CAR active site was located in a wideopen cleft,^4^ similar to cinnamoyl-CoA reductase 1 from *Sorghum bicolor* (PDB-code 5TQM).^14^

The hydride transfer from the NADPH is facilitated from its C4 position to a given substrate. To achieve a productive binding mode, the substrate must be located close to the C4 and activated by the enzyme. Substrate activation is facilitated by a proton relay system formed by Tyr844, Lys848 and putatively the ribityl backbone of the NADPH, as already described for other related short chain alcohol dehydrogenases like the cinnamoyl-CoA reductase 1 from *Sorghum bicolor*.^14^

## Minimal Substrate for Thioester Reduction

**Figure 3:**
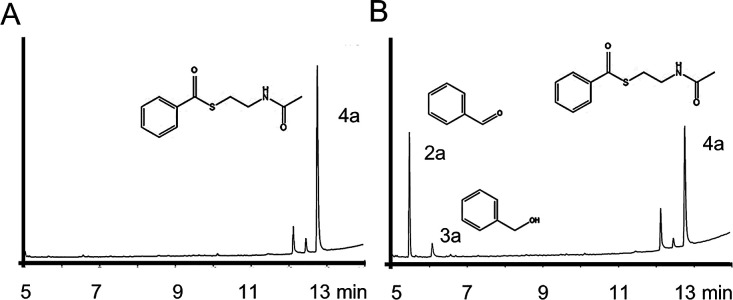
Reaction of (A) control (B) Thioester **4a** reduced to aldehyde **2a** and over-reduction to **3a** catalyzed by highly pure *Nc*CAR^Δ1–649^. Enzyme conc: 2.6 μM; Substrate conc. 10 mM; 20 h at 30 °C and pH 6.0.

The molecular function of CAR R-domains is, strictly speaking, the reductive cleavage of acylated phosphopantetheine (PPT) at the thioester moiety (Scheme 1B). In carboxylic acid reductions, the acyl moiety itself showed no interactions with the R-domain, when benzoyl-phosphopantetheine—the surrogate of **1a** (Scheme 1)—was modeled into bacterial R-domains.^4^ This led to the hypothesis that the interactions with the PPT arm might trigger substrate binding in the R-domain. We aimed to determine whether interactions of the thioester unit itself would suffice, however, neither *Nc*CARwt nor *Nc*CAR^Δ1–649^ reduced any of the 21 tested thioesters (SI, Table S3).^15^ This confirms early results with *Nc*CAR purified from its natural host^13^ and recombinant *Sr*CAR constructs, that failed to reduce thiobenzoate.^4^ Clearly, a compound must consist of more than only the thioester functionality. We next investigated a surrogate of **1** (Scheme 1), benzoyl-SNAc {**4a,** [S-(2-acetamidoethyl) benzothioate]}). This compound was readily reduced to **2a** (43% conversion), leading to the conclusion that the amide functionality connected via a C2 linker is the key to evoke substrate affinity (Figure 3). No **2a** was detected in control reactions with *Nc*CAR^Δ550–1052^ or full-length *Nc*CAR^Y844A^.

**Figure 4:**
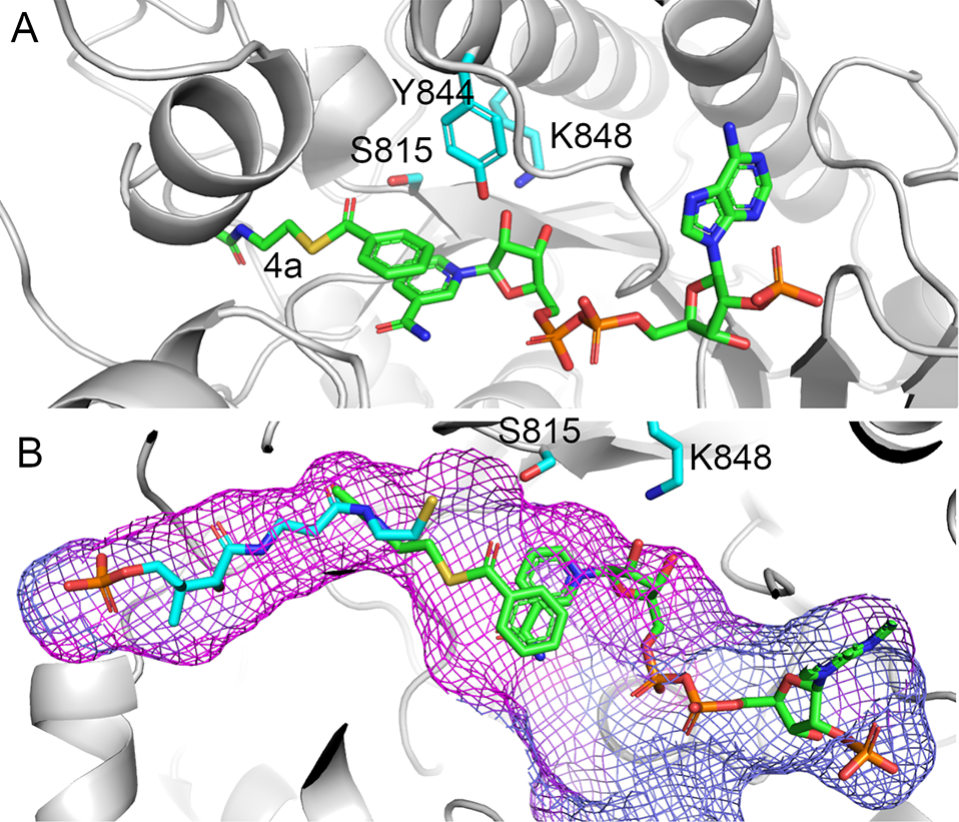
Proposed substrate binding in the *Nc*CAR R-domain active site (A) A putative productive ternary complex with NADPH and the minimal substrate **4a** (green). (B) phosphopantetheine (cyan) modeled into the tunnel of the *Nc*CAR R-domain.

This experimental result is supported by *in silico* modeling. To reduce the thioester, a ternary complex consisting of the enzyme, NADPH, and **4a** must be formed. To analyze putative productive binding modes that we have determined as minimal thioester-derived substrate, docking studies of **4a** to *Nc*CAR:NADPH were performed (Figure 4).

In this binding mode, the carbonyl carbon of the minimal substrate is located 4 Å from the NADPH C4 with a Bürgi–Dunitz angle of 105° between the carbonyl oxygen, carbonyl carbon and the C4 of the NADPH, indicating that the overlap of the NADPH-HOMO with the thioester-LUMO is given in this binding mode and that it therefore can be considered as a productive binding mode. Ser815 is described to be highly conserved in fungal CARs and short chain dehydrogenases/reductases (SDRs).^14^ In bacterial CARs, a Thr at this position is taking the role of activating the carbonyl moiety.^6^ As expected for a productive binding mode, an interaction is indicated between Ser815 and the carbonyl oxygen of **4a** in our docking. The acetyl-cysteamine moiety is buried (Figure 4A), indicating that in the full-length *Nc*CAR the benzoyl-intermediate **1a** is entering the active site via this tunnel. In full-length *Nc*CAR, the tunnel accommodates the phosphopantetheinyl moiety, as shown by a model of the reductase domain containing the prosthetic group, which is depicted in Figure 4 panel B in overlay with the docking mode of **4a**.

The phosphate moiety is located at the C-terminal end of the α helix formed by the residues Trp954 to Tyr964. The docking mode of **4a** and the model of phosphopantetheine in the tunnel are in good agreement. This indicates that a productive ternary complex for thioester reduction can be formed by NADPH entering the active site from one direction while the **1a**-intermediate enters from the opposite direction and occupies the tunnel (Figure 4). A domain movement might be necessary to open the tunnel to allow the tethered substrate to enter.

**Figure 5:**
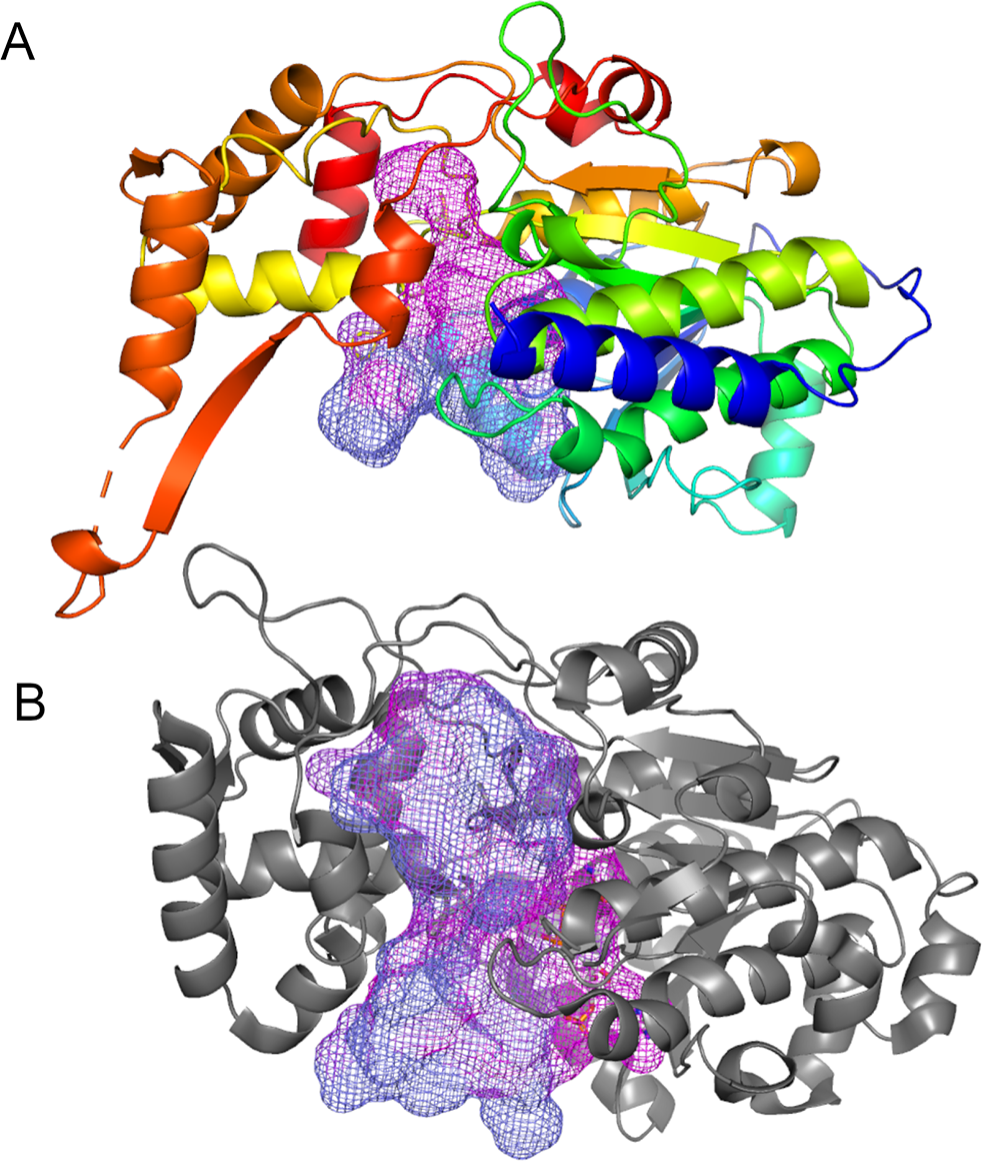
Comparison of the active site cavities of *Nc*CAR and *Sr*CAR R-domains. The cavities are depicted as mesh, coloring is according to Figure 2. (A) Cavity harboring the NADPH binding site, active site and putative phosphopantetheine linker tunnel in *Nc*CAR. The *Nc*CAR R-domain is rainbow colored from *N*-terminus (blue) to *C*-terminus (red), (B) Active site cleft of *Sr*CAR.

CARs are divided in five types (I–V).^6^ To elaborate the similarities and differences to other CARs, we superimposed the structure of *Nc*CAR R-domain (type III) to the *Sr*CAR PCP-R-didomain (PDB-code 5MSP_A) as a representative of a type I CAR that was reported to catalyze two-electron reduction of carboxylic acids (SI, Figure S4). A core alignment covering 312 of 396 residues with a RMSD of 2.1 Å and a sequence identity of 20.2% was achieved.^16^ The seven stranded parallel β sheet that forms the core of the Rossman fold, in combination with the respective helices that flank the sheet, are conserved. Less conserved is the *C*-terminal subdomain that is composed of helices and loops (SI, Figure S4B). Especially the residues Glu987 to Pro1019 of *Nc*CAR do not have a structural counterpart in *Sr*CAR. The respective structural elements from *Nc*CAR and *Sr*CAR (Leu1119 to Thr1149) are depicted in the SI, Figure S4C. In contrast to the *Nc*CAR active site with attached tunnel for the phosphopantetheine linker (Figure 4), a broad active site cleft is described for *Sr*CAR. The differing active site shapes of *Nc*CAR and *Sr*CAR R-domains are shown in Figure 5.

**Figure 6:**
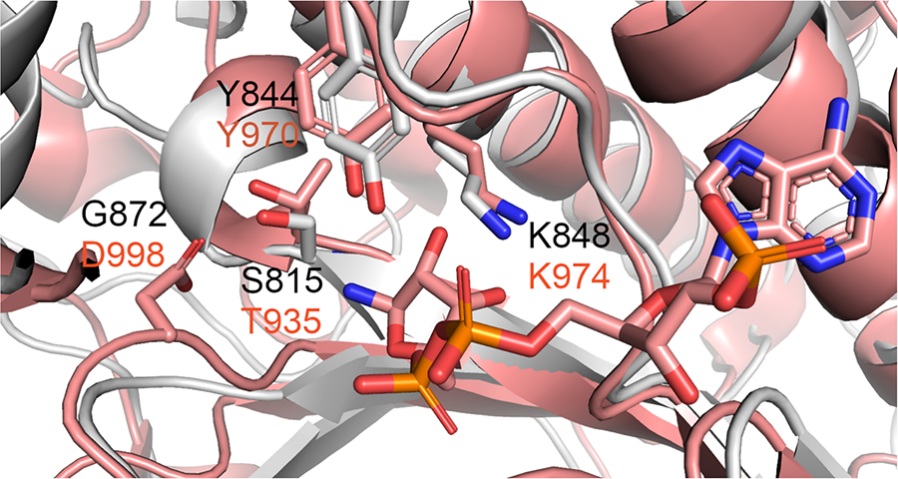
Catalytic residues in *Nc*CAR (gray) and *Sr*CAR (salmon) R-domain. Catalytic active site residues and the cofactor are depicted as sticks.

The different geometries of the cavities indicate that substrate binding and dynamics of the R-domain in *Nc*CAR and *Sr*CAR does differ to some extent. The catalytic residues in *Sr*CAR and *Nc*CAR are largely conserved (compare Figure 4) as described for cinnamoyl-CoA reductase 1 from *Sorghum bicolor*. Lys is interacting with the NADPH ribityl moiety and Tyr is interacting with the ribityl moiety in both CARs (Figure 6). Thr935 in *Sr*CAR corresponds to Ser815 in *Nc*CAR.

## Carbonyl Reduction Capacity of the R-Domain

The R-domain of *Nc*CAR is structurally similar to SDRs. Notably, incubation of **4a** with highly pure *Nc*CAR^Δ1–649^ gave not only **2a** but also benzyl alcohol **3a** (Figure 3), necessitating a thorough investigation of why and how the R-domain of *Nc*CAR is capable of catalyzing four-electron reduction.

**Figure 7:**
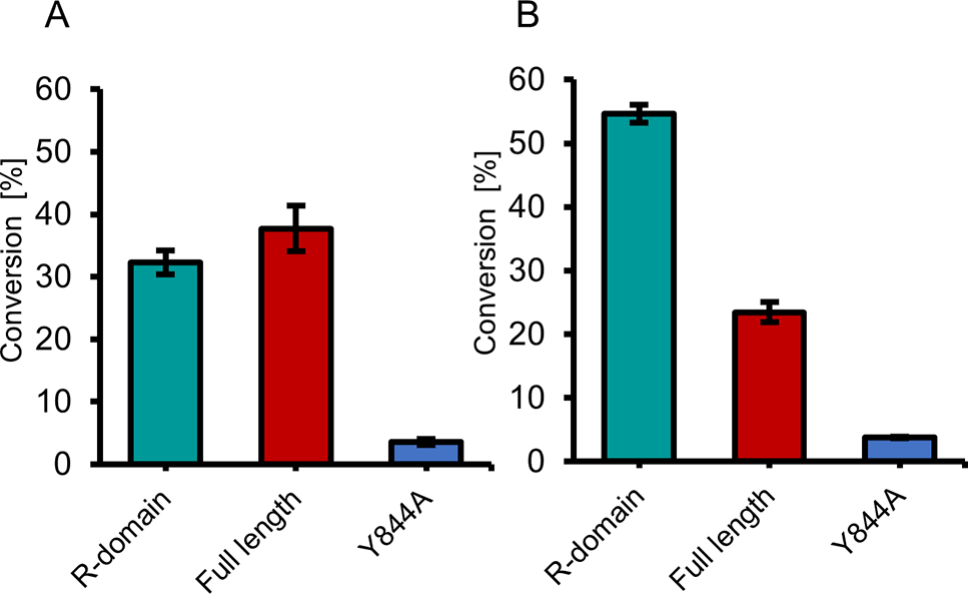
Reduction of (A) benzaldehyde (**2a**) and (B) octanal (**2b**). Enzyme conc: 8–9 μM; Substrate conc. 10 mM; 20 h at 30 °C and pH 6.0.

We subjected benzaldehyde (**2a**) and the simple aromatic ketone acetophenone (**5a**) to cell-free extract (CFE) of *Nc*CAR^Δ1–649^ producing *E. coli*. *Nc*CAR^Δ1–649^ was indeed able to reduce **5a** (73% conversion) to the secondary alcohol **6a**, while the A-domain alone (*Nc*CAR^Δ550–1052^) and a full-length *Nc*CAR variant with inactivated reductase domain (*Nc*CAR^Y844A^)^17^ were not (SI, Table S5). Reduction of **2a** to **3a** was observed in all CFE samples due to host background (SI, Figure S6);^18^ therefore, further experiments were carried out with purified *Nc*CAR variants (SI, Figure S7). Carbonyl reduction of **2a** and octanal (**2b**) was investigated with 0.4 mg·mL^–1^ of highly pure *Nc*CAR^Δ1–649^ and 1 mg·mL^–1^ of full-length enzymes in order to apply equimolar amounts of R-domain. Both the isolated R-domain and wild-type full-length *Nc*CAR catalyzed the reduction of aldehyde to alcohol, whereas inactivated reductase domain variant *Nc*CAR^Y844A^ did not. 32% of **2a** and 54% of **2b** was reduced by the R-domain alone (Figure 7).

Moving from aldehydes to ketones, both the full-length CAR and isolated R-domain produced secondary alcohols **6a**–**c** from the respective ketones **5a**–**c** in 10–20% yield (SI, Figure S8).

To analyze the conservation of the active site residues within this protein family, all sequences with a sequence similarity higher than 53% were retrieved from the NCBI and aligned using ClustalO (date February 2022). The grade of conservation and the proposed role of the residues within the first shell of the active site of the *Nc*CAR reductase domain are summarized in Table 1.

**Table 1:** Active Site Composition of the Reductase Domain of *Nc*CAR

residue	role in catalysis	conservation (%)
Ser815	activation of carbonyl oxygen	100
Tyr844^a^	proton relay system	100
Lys848^a^	proton relay system	100
Glu888	interaction with NADPH amide	100
Gln873	interaction with NADPH amide	100
Val874	hydrophobic interaction with nicotine amide	100^b^ Val/Ile
Val871	hydrophobic interaction with nicotine amide	100^b^ Val/Ile
Ile696	hydrophobic interaction with nicotine amide	100^b^ Ile/Leu
Phe780	active site shaping	100

a Tyr and Lys are essential residues for CAR activity.^6,17^

b Val, Ile, and Leu are considered as functionally invariant at this position.

**Figure 8:**
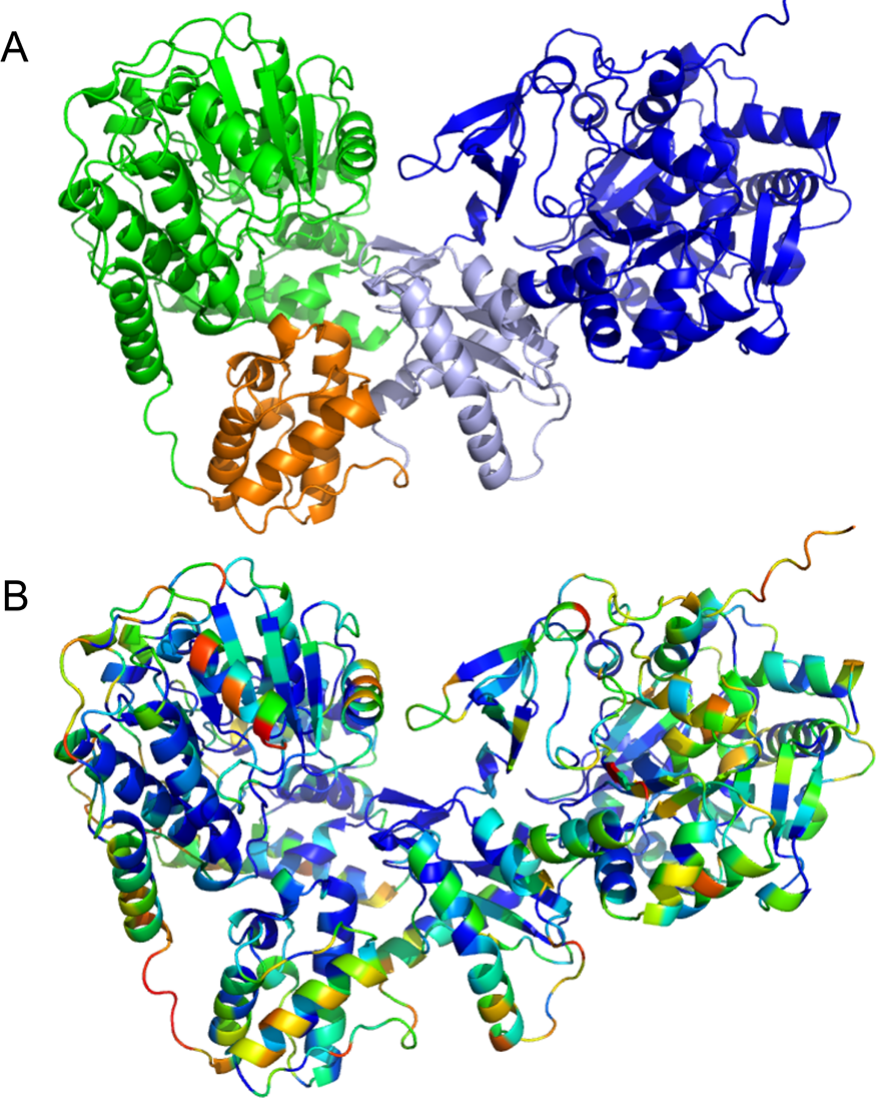
*Nc*CAR AlphaFold model. (A) Full-length model colored by its domains: A_core_ in blue, A_sub_ in pale blue, T in orange and R in green, (B) colored according to the sequence conservation score with blue regions being conserved and red regions variable (Sequence Alignment in Supporting Information).

The polar residues predicted to take part in the reaction were found to be highly conserved. Also, for the nonpolar residues that form a hydrophobic region where the nicotine amide is situated, only variations from Val to Ile or Ile to Leu were observed, i.e., variations that do not alter the properties of the enzyme at this position. To visualize the distribution of variation in the overall *Nc*CAR, an evolutionary conservation score was projected onto the full-length model generated by AlphaFold 2 (Figure 8).

In ATP limited conditions, the CAR itself may contribute to aldehyde reduction. Bacterial SDR-like enzymes were indeed reported to perform 4 e^–^-reductions,^20^ and so were terminal domains of fungal non-ribosomal peptide synthetases.^21^ Up to now, detected alcohols in the context of CAR reductions were primarily assigned to host-background reactivities. To minimize aldehyde depletion, knockout strains^22^ like the *E. coli* RARE strain^23^ were developed to serve as a platform for aldehyde synthesis with CARs. Over-reduction to alcohol was still detected,^24^ and seemed to be strongly substrate dependent. While <2% of benzylalcohol **3a** were found in full length *Nc*CAR mediated cell free reductions,^25^ > 30% of 3-nitro-benzylalcohol and 4-cyano-benzylalcohol accumulated when the respective acids were treated with *Nc*CAR in the presence of *in vitro* cofactor recycling components under the very same conditions. Strikingly, 97% of monomethylterephthalate was converted to the respective alcohol, which was previously unexplicable.^26^ The impact of CAR mediated over-reduction was assessed by direct comparison of aldehyde and acid reduction using an *in vitro* assay. While the specific activity of full length *Nc*CAR for benzaldehyde was only 1% of that for benzoic acid, the activity for octanal reduction was 0.11 μmol min^–1^ mg^–1^ as compared to 0.26 μmol min^–1^ mg^–1^ for octanoic acid, which is more than 40%. Strikingly, full length *Mycobacterium marinum* CAR (*Mm*CAR)^24^ activities for aldehyde reduction amounted to 16% and 66% of benzoic acid and octanoic acid reduction, respectively (SI, Figure S9).

Our results show that CARs R-domains contribute significantly more to undesired alcohol accumulation in the course of aldehyde syntheses than anticipated and that aldehyde reduction potential is strongly substrate dependent.

For bacterial CARs, it was reported that the highest degree of conservation is found for the reductase domain, while the A- and T- domains are less conserved.^4^ Also in fungal CARs, the highest conservation can be observed in the reductase domain, especially the catalytic residues and the residues forming the hydrophobic core are highly conserved. AlphaFold models of the full-length *Nc*CAR suggest a close arrangement of subdomains forming a compact structure (Figure 8A). The putative interfaces between the individual domains are highly conserved in this protein family (Figure 8B). The respective residues at the interfaces show a conservation of more than 90% (SI, Figure S11). This indicates the importance of the interface residues to ensure a functional domain interplay.

The representation of conserved residues indicates the formation of different clusters. Conserved regions are associated with the catalytic function of the respective subunit. Besides the interface residues, the hydrophobic core and the beta-sheets are highly conserved in the reductase domain. Domain swapping has been conducted with CARs and closely related non-ribosomal peptide synthases (NRPSs) as an enzyme engineering strategy.^4,27^ Our findings underline the importance of domain interface compatibility for engineering of multidomain enzymes. Furthermore, our results suggest the most crucial interface positions, that must match to ensure a functional full-length enzyme.

## Conclusion

We determined the first experimental structure of a reductase domain from a fungal CAR and evaluated its activity on a range of putative substrates. This structure is valuable for building more reliable models of fungal CARs^7^ and creates a basis for rational enzyme engineering. Substrate recognition in this subdomain is based on the presence of the acetyl-cysteamine moiety and not on the presence of a thioester in the putative substrate. The presence of the acetyl-cysteamine moiety is essential for substrate recognition in thioester reduction, as it mimics the phosphopantetheine arm, which is the natural prosthetic group of CARs. Other thioesters lacking this group were not accepted as substrates for thioester reduction to aldehyde. We identified a tunnel in the R-domain that putatively harbors the PPT arm, which is in good agreement with docking experiments conducted with the minimal substrate **4a**. These results indicate that CARs do not rely on the ATP-dependent activation of a substrate but that this step can be circumvented by employing SNAC-esters. This strategy proved to be a versatile tool for biotransformations with non-ribosomal peptidesynthases.^28^

Substrate screening experiments revealed that the presence of the acetyl-cysteamine moiety is strictly necessary for the reduction of thioesters to aldehydes, while it is not required for the reduction of carbonyl compounds like benzaldehyde or benzophenone to the respective alcohols. This aldehyde over-reduction was previously attributed to the host strain background, but our results indicate that alcohol formation is an intrinsic side activity of CAR R-domains. Ultimately, aldehyde overreduction cannot be attributed exclusively to the host background and its extent is substrate dependent.

## Supporting Information Available

Additional tables and figures, experimental details (PDF)
Sequence Alignment (ALN)
