# Supplementary material for: Structure of the Reductase Domain of a Fungal Carboxylic Acid Reductase and Its Substrate Scope in Thioester and Aldehyde Reduction
Source: ACS Catal. 2022 Dec 6;12(24):15668–74. doi: 10.1021/acscatal.2c04426 (PMC10168641; doi:10.1021/acscatal.2c04426)
Supplement: Supplementary file 1 — cs2c04426_si_001.pdf [file cs2c04426_si_001.pdf]

Supporting Information

Structure of the Reductase Domain of a fungal Carboxylic Acid Reductase and its Substrate Scope in Thioester and Aldehyde Reduction

Bastian Daniel,[§,$,‡] Chiam Hashem,[§,&,‡] Marlene Leithold,[§,$] Theo Sagmeister,[$] Adrian Tripp,[c] Holly Stolterfoht-Stock,[§] Julia Messenlehner,[c] Ronan Keegan,[d] Christoph K. Winkler,[e] Jonathan Guyang Ling,[#] Sabry H.H. Younes,[f,g] Gustav Oberdorfer,[c] Farah Diba Abu Bakar,[#] Karl Gruber,[§,$,a,b] Tea Pavkov-Keller,*[§,$,a,b] Margit Winkler*[§,&]

[§] acib - Austrian Center of Industrial Biotechnology, Krenngasse 37, 8010 Graz, Austria

[$] Institute of Molecular Biosciences, University of Graz, Humboldtstraße 50, 8010 Graz, Austria

[&] Institute of Molecular Biotechnology, Graz University of Technology, Petersgasse 14, 8010 Graz, Austria

[#] Department of Biological Sciences and Biotechnology, Universiti Kebangsaan Malaysia, 43600 Bangi, Selangor, Malaysia

[a] BioHealth Field of Excellence, University of Graz, 8010 Graz, Austria

[b] BioTechMed-Graz, Graz, Austria

[c] Institute for Biochemistry, Graz University of Technology, Petersgasse 12, 8010 Graz, Austria

[d] Rutherford Appleton Laboratory, Research Complex at Harwell, UKRI-STFC, Didcot OX11 0FA, United Kingdom

[e] Institute of Chemistry, University of Graz, Heinrichstraße 28, 8010 Graz, Austria

[f] Department of Chemistry, Faculty of Science, Sohag University, Sohag 82524, Egypt

[g] Department of Biotechnology, TU Delft, Van der Maasweg 9, 2629HZ Delft

[‡] These authors contributed equally

*margitwinkler@acib.at, margit.winkler@tugraz.at; tea.pavkov@uni-graz.at

Table of Contents

1. General Material 3
2. Expression of full-length *Nc*CAR^{wt} and cleavage to subdomains 3
3. Cloning and Expression of the R-domain of *Nc*CAR (*Nc*CAR$^{\Delta 1\text{-}649}$) and control strains 3
4. Protein crystallization 4
5. Determination of oligomerization state of full length *Nc*CAR 8
6. Chemical Synthesis 8
   6.1. Synthesis of *S*-benzoyl-*N*-acetylcysteamine[12] 8
   6.2. Synthesis of thioester library 9
7. Biotransformations 10
   7.1. Reduction of **1a** & **4a** with CFE 11
   7.2. Reductions with purified enzymes 12
   7.3. *In-vitro* comparison of aldehyde and acid reduction by full length CARs 14
8. Phylogeny 15
9. References 16

1. General Material

Standard reagents were obtained from VWR, Sigma-Aldrich (Vienna, Austria) or Roth GmbH & Co. KG (Karlsruhe, Germany) with the highest purity available. Restriction enzymes were obtained from Thermo Scientific (St. Leon Rot, Germany).

2. Expression of full-length *Nc*CAR^{wt} and cleavage to subdomains

The expression of *Neurospora crassa* CAR (Q7RW48) was conducted in *Escherichia coli* BL21 Star^{TM} (DE3) cells using the pETDuet1 vector as previously described by Schwendenwein *et al*. with *Ec*PPTase (CAQ31055.1) at multiple cloning site (MCS) 1 and the *Nc*CAR sequence in MCS2 (*Nc*CAR^{wt}, Table S1).[1] Variant *Nc*CAR^{Y844A} was cloned and produced as described previously.[2] To facilitate the purification of individual domains, a WELQut protease cleavage site was introduced after residue 648 to create the construct AT-WELQ-648-R. After size exclusion chromatography, the construct was diluted to a concentration of 1 mg/mL in Tris/HCl-buffer (0.1 M, pH 8.0). 1 µL of WELQut protease (5 U/µL Thermo Fischer Scientific Inc., Waltham, USA) was used per 0.53 µg of cleavable protein. The digestion was performed for 26.5 h at 16 °C and 500 rpm. Subsequently, the domains were separated via size exclusion chromatography and used for crystallography.

3. Cloning and Expression of the R-domain of *Nc*CAR (*Nc*CAR$^{\Delta 1\text{-}649}$) and control strains

A gene for the construct corresponding to the cleavage product of the AT-WELQ-648-R was cloned with an *N*-terminal His-tag and a TEV-protease cleavage site by addition of MSYYHHHHHHHHHHDYDIPTTENLYFQGA to the respective subunit. The resulting vector designated pETDuet:*Ec*PPTase_*Nc*CAR_R_domain_Nterm_10xHisTag was used for transformation of electrocompetent *E. coli* BL21 Star^{TM} (DE3) and the gene was expressed as described above. 6.0 g cells were lysed by sonication in 40 mL sodium phosphate buffer (20 mM, pH 7.4) containing 500 mM NaCl and 10 mM imidazole. Cell debris was removed by centrifugation using an Avanti J-26-XP centrifuge (Beckman Coulter, Brea, USA) equipped with a JA 25.50 rotor (Beckman Coulter, Brea, USA) for 30 minutes at 4 °C at 16,000 rpm. The supernatant was filtered through a 0.45 µM syringe filter and applied to a 5 mL HisTrap Fast Flow column (GE Healthcare, Chicago, USA) using an ÄKTA system (Cytivia, Marlborough, USA) and eluted with sodium phosphate buffer (20 mM, pH 7.4) containing 500 mM NaCl and 500 mM imidazole. R-domain containing fractions were concentrated to a final volume of 28 mL and a concentration of 3 mg/mL. 400 µL TEV protease (kindly provided by Tamara Berger, 1.5 mg/mL) and 21 mg DTT were added and incubated for 3 h at 22 °C. The cleaved protein was dialyzed in 5 L of sodium phosphate buffer (20 mM, pH 7.4) containing 500 mM NaCl, 10 mM imidazole and 1 mM DTT overnight. The dialyzed protein solution was loaded to a Ni-NTA column and treated as described above. The cleaved R-domain, corresponding to *Nc*CAR$^{\Delta 1\text{-}649}$ with two additional N-terminal residues GA was eluted using sodium phosphate buffer (20 mM, pH 7.4) containing 500 mM NaCl, concentrated and loaded to a HiLoad ® 16/600 Superdex 200 pg size exclusion column (GE Healthcare, Chicago, USA) and eluted with Tris/HCl buffer (10 mM, pH 7.4) containing 150 mM NaCl at a flow rate of 0.5 mL/min. The separately expressed R-domain was used for crystallization trials and activity assays.
Similarly, the single A domain of *Nc*CAR was cloned and produced (*Nc*CAR$^{\Delta 550\text{-}1052}$) as a control (Table S1).

Table S1. ***Neurospora crassa*** **CAR constructs with calculated molecular weight.**

Construct	Abbr.	Amino acid residues	MW [kDa]
*Nc*CAR$^{\Delta 1\text{-}649}$	R-domain	649-1052	45
*Nc*CAR^{wt}	Full-length, wild type*	1-1052	121
*Nc*CAR$^{\Delta 550\text{-}1052}$	A-domain	1-549	62
*Nc*CAR^{Y844A}	Full-length, variant unable to catalyze thioester reduction	1-1052	121

**Nc*CAR^{wt} with WELQut site has an insertion of 3 amino acids between Asn647 and Gln648.

4. Protein crystallization

Protein crystallization was conducted by vapor diffusion technique using Swissci plates (Swissci AG, Neuheim, Switzerland) and an Oryx8 robot (Douglas Instruments, Berkshire, UK) employing the Index screen (Hampton Research, Aliso Viejo, USA). Crystals were grown by mixing 0.5 µL ammonium sulfate (0.2 M), BisTRIS (0.1 M, pH 5.5) and 25 % PEG 3350 with 0.5 µL *Nc*CAR R-domain (18.00 mg/mL) generated via WELQut protease digestion as described above on a Swissci triple well plates (Molecular Dimensions, Holland, USA) using the sitting drop method with a reservoir volume of 33 µL. Crystals appeared within 6-12 weeks and were frozen in liquid nitrogen without cryo protection for diffraction experiments. A data set with a resolution of 2.3 Å was collected at the ID23-2 beamline of the ESRF in Grenoble (France) at 100 °K. Data processing was performed with the XDS program package.[3] Unit cell parameters and assigned space groups as well as data statistics are shown in Table S2.

Table S2. Data collection and refinement statistics

PDB-code	8AEP
Resolution range	41.48 - 2.3 (2.382 - 2.3)
Space group	P 1 21 1
Unit cell	56.235 137.487 66.513 90 112.333 90
Total reflections	157581 (16017)
Unique reflections	41354 (4128)
Multiplicity	3.8 (3.9)
Completeness (%)	99.58 (99.78)
Mean I/sigma(I)	6.68 (1.93)
Wilson B-factor	21.01
R-merge	0.2059 (0.7506)
R-meas	0.2396 (0.8712)
R-pim	0.1212 (0.438)
CC1/2	0.219 (0.631)
CC*	0.599 (0.88)
Reflections used in refinement	41284 (4128)
Reflections used for R-free	2039 (197)
R-work	0.2020 (0.2685)
R-free	0.2616 (0.3228)
CC(work)	0.928 (0.754)
CC(free)	0.878 (0.619)
Number of non-hydrogen atoms	6959
macromolecules	6206
ligands	12
solvent	741
Protein residues	792
RMS(bonds)	0.015
RMS(angles)	1.81
Ramachandran favored (%)	96.68
Ramachandran allowed (%)	3.32
Ramachandran outliers (%)	0.00
Rotamer outliers (%)	0.15
Clashscore	2.11
Average B-factor	28.14
macromolecules	27.77
ligands	41.76
solvent	31.00

The solvent content was estimated based on the calculated Matthews coefficient.[4] Phasing was performed using a truncated ensemble made from distant homologues employing the CCP4mg-MrBUMP task.[5] Phases were improved by SHELXE[6] to produce a c-alpha trace followed by model building employing Buccaneer.[7] Refinement was conducted by repetitive

rounds of REFMAC in CCP4 and manual model building in COOT.[4,8] A model has been deployed with PDB-code 8AEP.

SAXS data were collected on the BioSAXS BM29 beamline (ESRF, Grenoble) equipped with the Pilatus3 2M detector (Dectris, Baden-Dättwil, Switzerland) and a sample-to-detector distance of 2.867 m. An X-ray wavelength of 0.99 Å (12.5 keV) was used for the measurement. Data were collected on 3 concentrations ranging from 1 to 5 mg/ml *Nc*CAR-R in with 10 mM Tris/HCl buffer pH 7.4 containing 150 mM NaCl. Buffer measurements were performed between each sample. For each data collection, 20 frames of 100 ms were collected. Scattering collected on the pure buffer was subtracted from that of the sample, and the resulting curves were normalized to the protein concentration.

The data processing was performed within the ATSAS package.[9] The program CRYSOL was used to compute the theoretical curves from the atomic structures.[10] Volume fractions of the components of the oligomeric mixtures (monomer + crystallographic dimer) were computed and fitted to the data using the program OLIGOMER.[11]

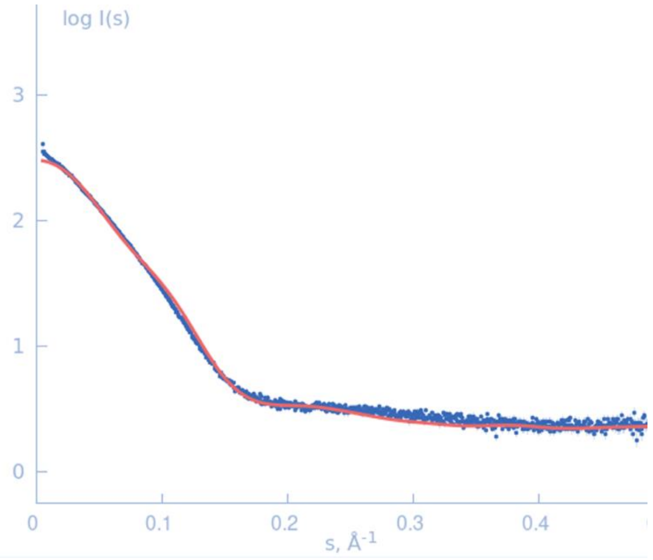

Figure S1. *SAXS-curve of the R-domain (blue) and best fit (red)*

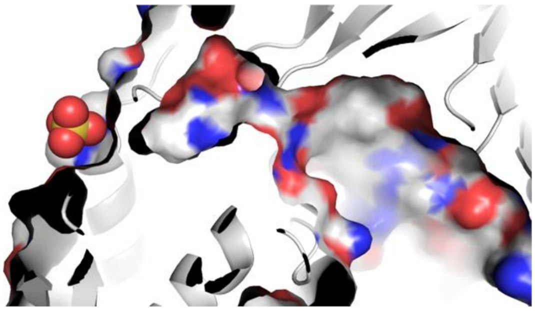

Figure S2. *Tunnel in the NcCAR that accommodates the active site. NADPH is hypothesized to enter via the broad opening while the PPT-linker enters via the tunnel. The phosphate group of PPT putatively occupies the sulfate-binding site in this case*

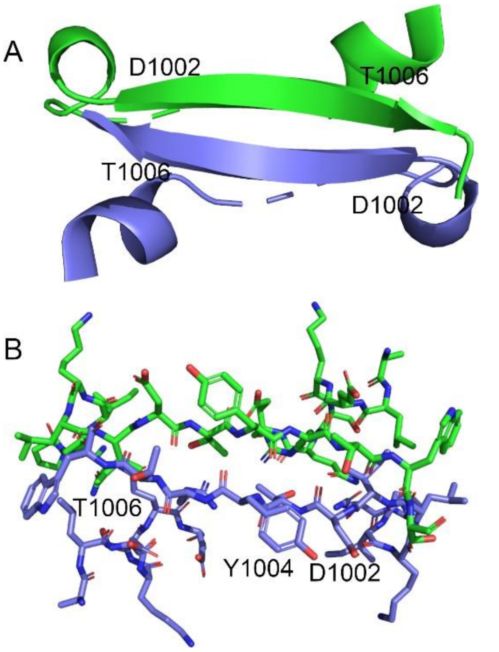

Figure S3. ***A)*** *Symmetrical beta-sheet between chain A and B in the asymmetric unit determined for the R-domain that is formed by Asp1002 to T1006.* ***B)*** *Stick representation of the shared beta-sheet between chain A and B. Asp1002A interacts with T1006B and respectively Asp1002B with T1006A, Y1004A in the centre of the sheet interacts with Y1004B.*

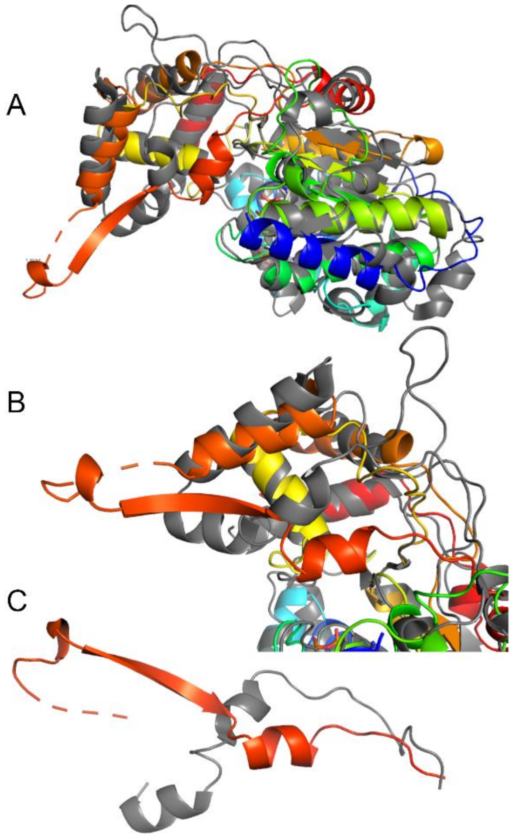

Figure S4. ***A)*** *Comparison of NcCAR R-domain and the SrCAR PCP-R-didomain (pdb code 5MSP_A). The R-domain is depicted as cartoon and colored by a blue to red gradient from the N- to C-terminus. The R-domain of SrCAR is depicted in gray.* ***B)*** *The C-terminal subdomain of R-domain that is responsible for the formation of the active site cleft in SrCAR and the respective tunnel in NcCAR.* ***C)*** *Structural elements with the highest variation between NcCAR and SrCAR; the respective beta-strand was found to form an antiparallel beta-sheet with the second chain in the asymmetric unit of the NcCAR R-domain (compare Figure S3). The adjacent helix is involved in the formation of the tunnel that is putatively harboring the phosphopantetheine moiety (compare Figure 3 and 5).*

5. Determination of oligomerization state of full length *Nc*CAR

*Nc*CAR expressed similarly to methods described in Section 3. *Nc*CAR was purified via IMAC with a 5 mL HisTrap Fast Flow column (GE Healthcare, Chicago, USA) using an ÄKTA system (GE Healthcare, Chicago, USA) and eluted with Tris/HCl buffer (20 mM, pH 7.4) containing 500 mM NaCl and 500 mM imidazole. Eluted *Nc*CAR was concentrated to 0.5 mL and loaded onto a HiLoad ® 16/600 Superdex 200 pg size exclusion column (GE Healthcare, Chicago, USA) and eluted with Tris/HCl buffer (20 mM, pH 7.4) containing 150 mM NaCl at a flow rate of 0.5 mL/min. Gel Filtration Standards (Bio-Rad, USA) were reconstituted in 0.5 mL deionized water, loaded and eluted as aforementioned (Figure S5). The molecular mass of the main peak eluting at 69 mL (Figure S5A) was determined to be 116 kDa, which corresponds to *Nc*CAR in its monomeric form. A peak eluting at approximately 58 mL retention volume is also comprised of *Nc*CAR according to gel electrophoreses and likely represents a trimer.

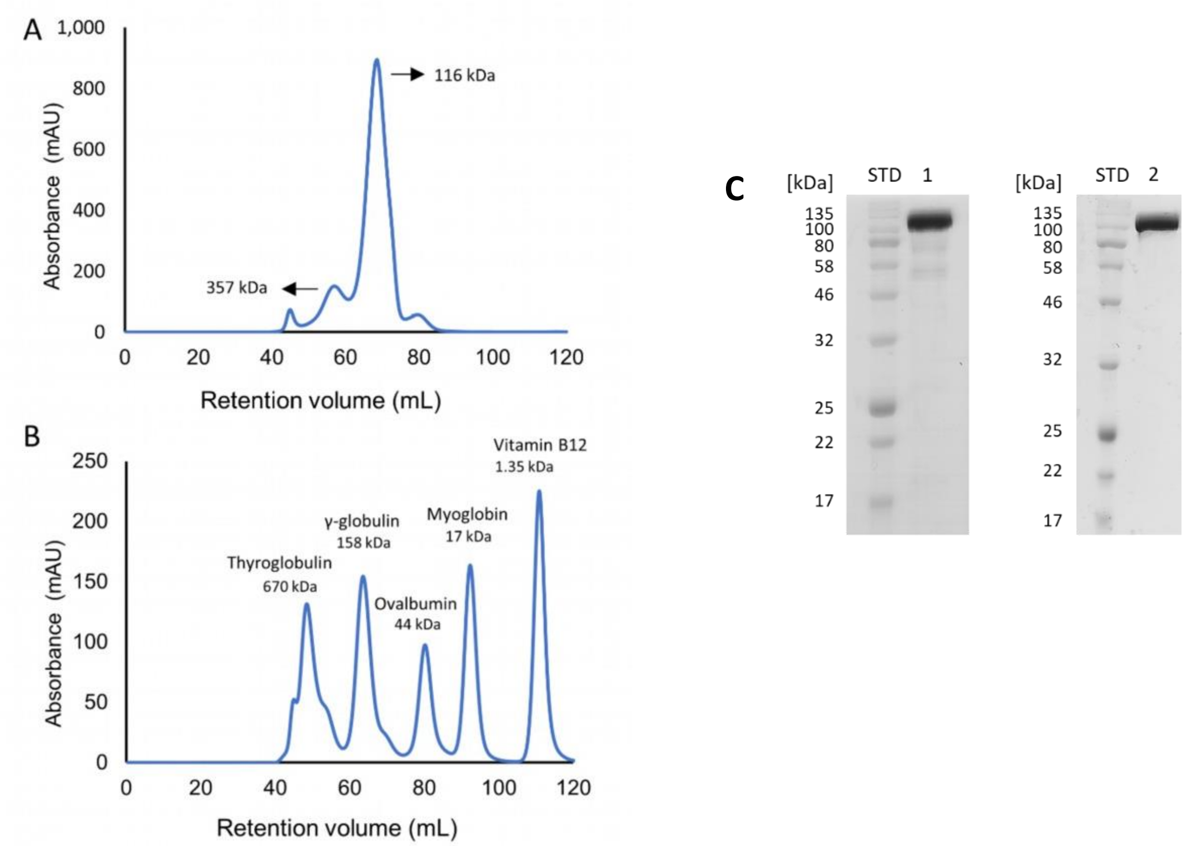

***Figure S5**. Size exclusion chromatography of **A)** full length NcCAR and **B)** Gel Filtration Standards (BioRad) **C)** SDS-PAGE profiles for the purification of full length NcCAR. **STD**: Prestained Protein Standard (New England Biolabs), **1**: peak eluted at 58 mL and **2**: peak eluted at 69 mL.*

6. Chemical Synthesis

6.1. Synthesis of *S*-benzoyl-*N*-acetylcysteamine[12]

Benzoyl chloride (0.5 mL, 4.5 mmol) was added slowly to a solution of *N*-acetylcysteamine (288 µL, 3 mmol) and triethylamine (835 µL, 6 mmol) in 20 mL diethyl ether at 0 °C, which led

to a white precipitate immediately. The reaction was quenched by adding saturated aqueous NH_4Cl (20 mL) and H_2O (40 mL) and extracted twice with ethyl acetate (80 mL each). The combined organic phases were concentrated *in vacuo*, which gave the solid product. For purification, the crude product was filtered and washed with ice-cooled ethyl acetate (5 mL). After drying at 60 °C, the crude product was purified by column chromatography (silica gel; hexane:EtOAc 1:10, TLC: Rf = 0.29) which yielded *S*-benzoyl-*N*-acetylcysteamine (0.54 g, 80%) as a white solid. ^{1}H NMR (300 MHz, DMSO-d_6), δ (ppm) = 7.9 (d, 2H, J = 1.2 Hz), 7.7 (t, 1H, J = 7.5 Hz), 7.6 (t, 2H, J = 7.5 Hz), 3.3 (t, 2H, J = 6.3 Hz), 3.1 (t, 2H, J = 6.6 Hz), and 2.5 (s, 3H). ^{13}C NMR (75 MHz, DMSO-d_6) δ (ppm) = 191.5, 172.2, 136.8, 133.4, 128.5, 126.8, 38.8, 27.9, and 21.1.

6.2. Synthesis of thioester library

The synthesis of the respective thioesters was described previously.[13] They are summarized in Table S3, with the corresponding GC-FID method (SI, Chapter 6).

Table S3 List of tested thioesters and corresponding GC-FID method.

Code	Compound	Chemical Formula	GC-Method
T1	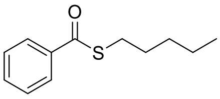	$C_{12}H_{16}OS$	A
T2	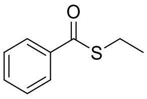	$C_9H_{10}OS$	A
T3	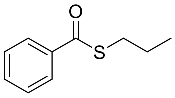	$C_{10}H_{12}OS$	A
T4	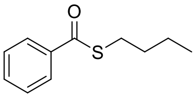	$C_{11}H_{14}OS$	A
T5	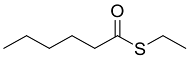	$C_8H_{16}OS$	C
T6	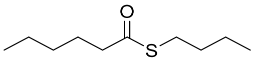	$C_{10}H_{20}OS$	C
T7	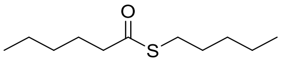	$C_{11}H_{22}OS$	C
T8	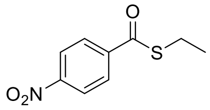	$C_9H_9NO_3S$	A
T9	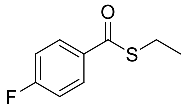	C_9H_9FOS	A
T10	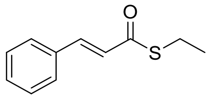	$C_{11}H_{12}OS$	A
T11	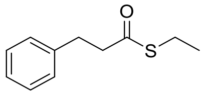	$C_{11}H_{14}OS$	A
T12	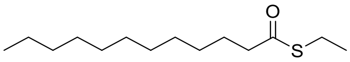	$C_{14}H_{28}OS$	D

T13	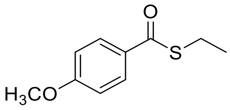	$C_{10}H_{12}O_2S$	A
T14	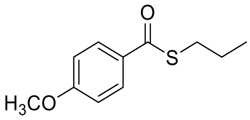	$C_{11}H_{14}O_2S$	A
T15	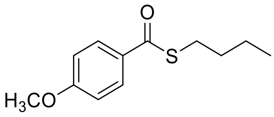	$C_{12}H_{16}O_2S$	A
T16	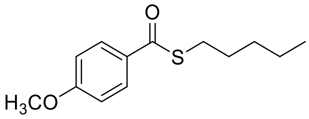	$C_{13}H_{18}O_2S$	A
T17	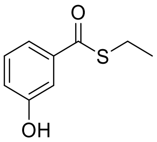	$C_9H_{10}O_2S$	A
T18	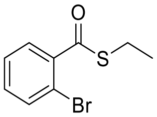	C_9H_9BrOS	A
T19	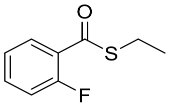	C_9H_9FOS	A
T20	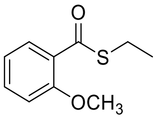	$C_{10}H_{12}O_2S$	A
T21	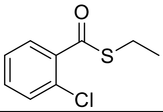	C_9H_9ClOS	A

7. Biotransformations

Biotransformations were carried out in triplicates using cell free extracts (CFE) as well as purified enzymes. Cell free extracts were diluted 1:10 (~10mg/mL) in 50 mM sodium phosphate pH 7.75 in a volume of 1 mL per reaction. Purified enzymes were thawed on ice and diluted to appropriate concentrations (8-9 µM) in 50 mM sodium phosphate pH 7.75. Reactions were initiated with 1 % DMSO and 10 mM NADPH at 30 °C for approximately 20 hours. After selected times, reactions were terminated with the addition of HCl (6 M). Subsequently, 500 µL of each bioconversion was vortexed for 2 min after addition of equal volumes of ethyl acetate to extract organic compounds. The organic phase was dried with Na_2SO_4 (approximately 10 % (w/v)) prior to transferring 200 µL into glass vials for GC analysis.

A Shimadzu 2010 Plus equipped with a flame ionization detector and a ZB-5 column (30 m, 0.25 µm, 0.32 mm, Agilent technologies) was used to analyze the reactions. Sample aliquots of 1 µL were injected in split mode (split ratio 10:1). Methods for each substrate group was adapted in order to reach clear separation of substrate and reduced product. Analytical

standards, as well as internal standard tetradecane (0.01 %), were used to determine analyte concentrations (Table S4).

Method A: Column temperature was increased from 60 °C to 320 °C at 20 °C/min. Injection temperature: 240 °C, detector temperature: 330 °C.
Method B: Column temperature was increased from 60 °C to 120°C at 5 °C/min, and from 120 °C to 320 °C at 20 °C/min. Injection temperature: 240 °C, detector temperature: 330 °C.
Method C: Column temperature was held from 70 °C for 10 min, then increased to 300 °C at 40°C/min. Injection temperature: 240 °C, detector temperature: 320 °C.
Method D: Column temperature was held at 70 °C for 4 min, then increased to 130 °C at 5 °C/min, and subsequently increased to 300 °C at 45 °C/min. Injection temperature: 240 °C, detector temperature: 320 °C.

Table S4 Substrates and corresponding products after reduction.

Abbr.	Substrate	Abbr.	Product	GC-Method
**2a**	Benzaldehyde	**3a**	Benzyl alcohol	A
**2b**	Octanal	**3b**	Octanol	D
**5a**	2-Nonanone	**6a**	2-Nonanol	D
**5b**	Acetophenon	**6b**	2-Phenylethanol	B
**5c**	4-Phenyl-2-butanone	**6c**	4-Phenyl-2-butanol	B
**4a**	N-(2-((1-phenylvinyl)thio)ethyl)acetamide	**2a/3a**	Benzaldehyde/Benzyl alcohol	A

7.1. Reduction of 1a & 4a with CFE

We wondered, whether typical short chain dehydrogenase substrates would be reduced; hence, we subjected the CFE from *E. coli* (Figure S6) clones expressing the R-domain, or the A-domain or the full-length Y844A variant (the latter two as controls) to the aromatic aldehyde **2a** and the simple aromatic ketone **5b**. Cofactor recycling, adapted from Strohmeier *et. al.*, was applied to provide enough NADPH.[14] Samples were analyzed by GC-FID (Table S5).

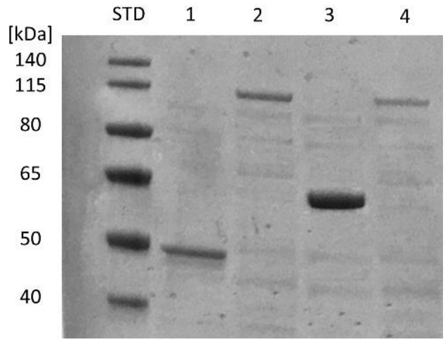

Figure S6. *Expression profile of Neurospora crassa CAR constructs in Escherichia coli cell free extract.* ***1****: R-domain (45 kDa),* ***2****: Full-length NcCAR^{wt} (121 kDa),* ***3****: A-domain (62 kDa) and* ***4****: Full-length Y844A variant (121 kDa). STD: Prestained Protein ladder (ThermoFisher)*

Table S5. Bioconversions of aromatic aldehyde and ketone with cell free extract.

		Analytical yield [%]	
**Construct**		**6b**	**3a**
*Nc*CAR$^{\Delta 1\text{-}649}$	R-domain	72.8 ± 0.2	76.4 ± 3.3
*Nc*CAR$^{\Delta 550\text{-}1052}$	A-domain	0.57 ± 0.01	70.3 ± 21.9
*Nc*CAR^{Y844A}	Full-length, variant unable to catalyze thioester reduction	0.10 ± 0.01	43.8 ± 3.8

*Nc*CAR R-domain reduced acetophenone **5b** to its corresponding alcohol **6b**. After 20 hours we observed more than 72% conversion.

Aldehydes are actually considered as final products upon CAR reactions, so we decided to investigate putative over reduction, which are reported frequently. Reduction of **2a** to **3a** by the R-domain was confirmed by GC-FID analysis, but not surprisingly, this product was also found in the cell free-extracts of our controls. To gain a better understanding, we must go one step further and purify our constructs to reduce *E. coli* background activities.

7.2. Reductions with purified enzymes

We used immobilized metal-affinity chromatography (on Ni-Sepharose) to eliminate *E. coli* background activities from the *Nc*CAR R-domain, the full-length *Nc*CAR^{wt}, and *Nc*CAR Y844A variant as a negative control. Buffer exchange was performed via size exclusion chromatography and approximately 3 µg of protein was applied on each lane of the SDS-polyacrylamide gel slab (Figure S7).

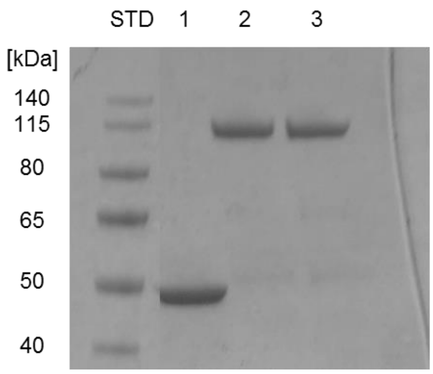

Figure S7. *Detection of IMAC-purified NcCAR constructs.* ***1:*** *R-domain (45 kDa),* ***2:*** *Full-length CAR^{wt} (121 kDa) and* ***3:*** *Y844A variant (121 kDa) were purified on Ni-Sepharose columns, approximately 3 µg of protein was applied on each lane.*

Ketone reduction was confirmed with purified R-domain for **5a**, **5b** and **5c** (Figure S8). Here, the R-domain produced approximately 18% of **6b**, and over 10% of both, **6a** and **6c**. Equimolar concentration of full-length enzyme showed much lower rate of conversions (8%) and further, theY844A variant did not reveal any conversions at all. To our knowledge, this is the first time that these 3 ketones have been reduced by a single domain of a fungal CAR.

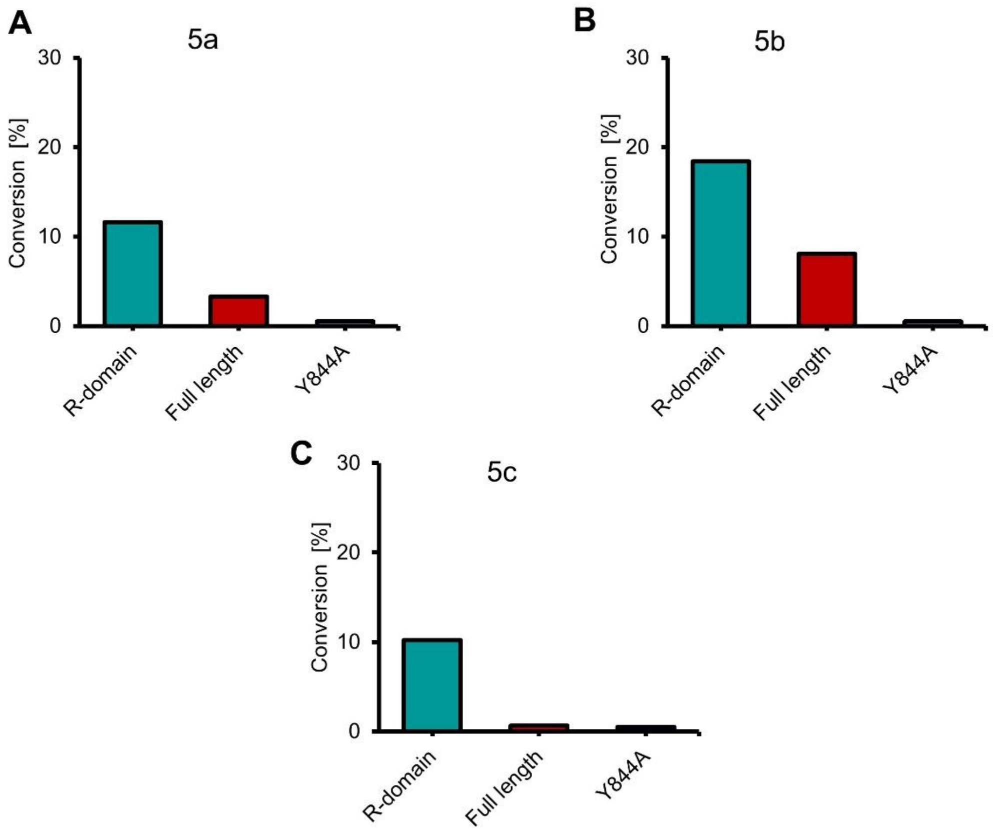

Figure S8. *Bioconversions of* ***A)****:* ***5a****,* ***B)****:* ***5b*** *and* ***C)****:* ***5c*** *after 20 hours of incubation with the R-domain, full-length CAR^{wt} and Y844A variant.*

7.3. *In-vitro* comparison of aldehyde and acid reduction by full length CARs

Specific activities of IMAC purified full length *Nc*CAR and the full length CAR from *Mycobacterium marinum* were determined as previously described.[1] The buffer was MES (100 mM, pH 6.5, 10 mM $MgCl_2$) and final substrate concentration was 10 mM in DMSO (5% v/v final conc.). ATP (1 mM) was present in all reactions (Figure S9).

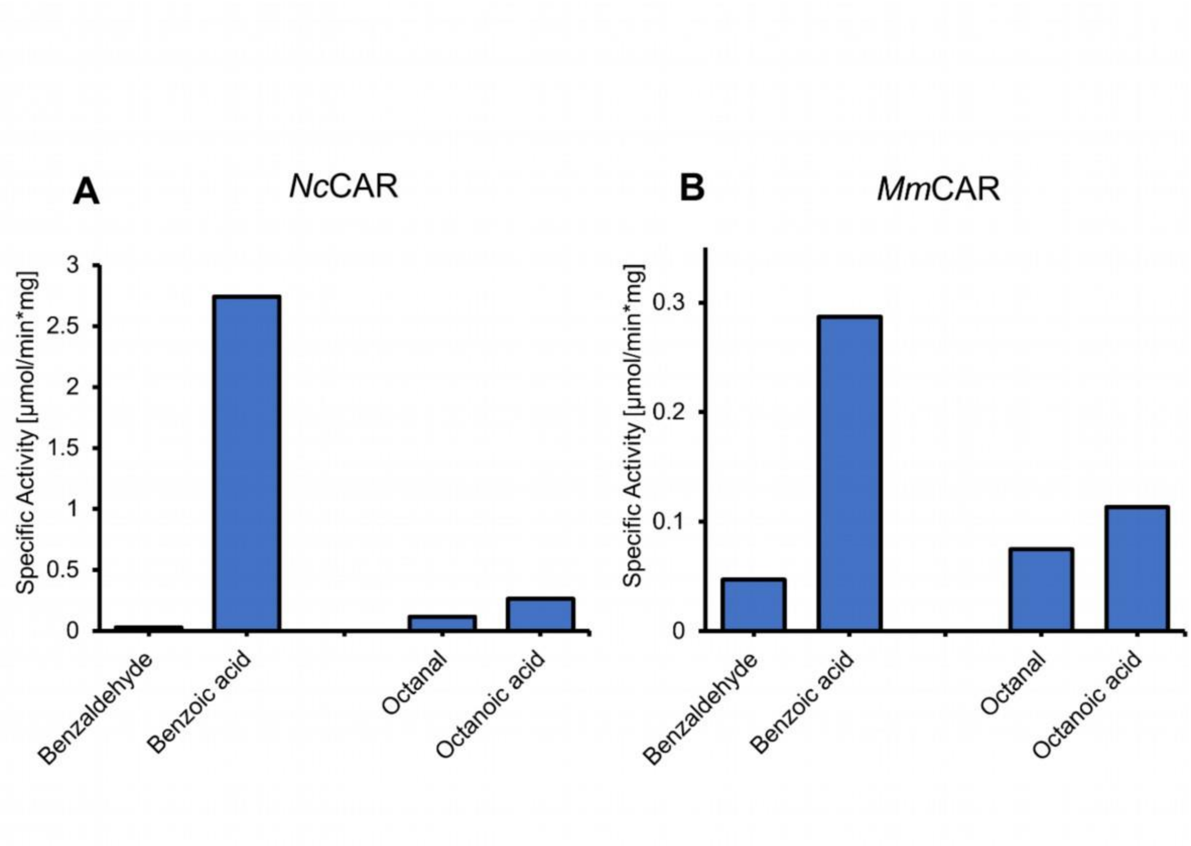

Figure S9. Specific activities of full length CARs from A) *N. crassa* (type III CAR) and B) *M. marinum* (type I CAR).

8. Phylogeny

A set of sequences was retrieved from the NCBI and aligned with ClustalO. The respective alignment can be retrieved as Supplemental file S1. In Jalview a neighbor joining tree (NJ) was created that is depicted in Figure S10.

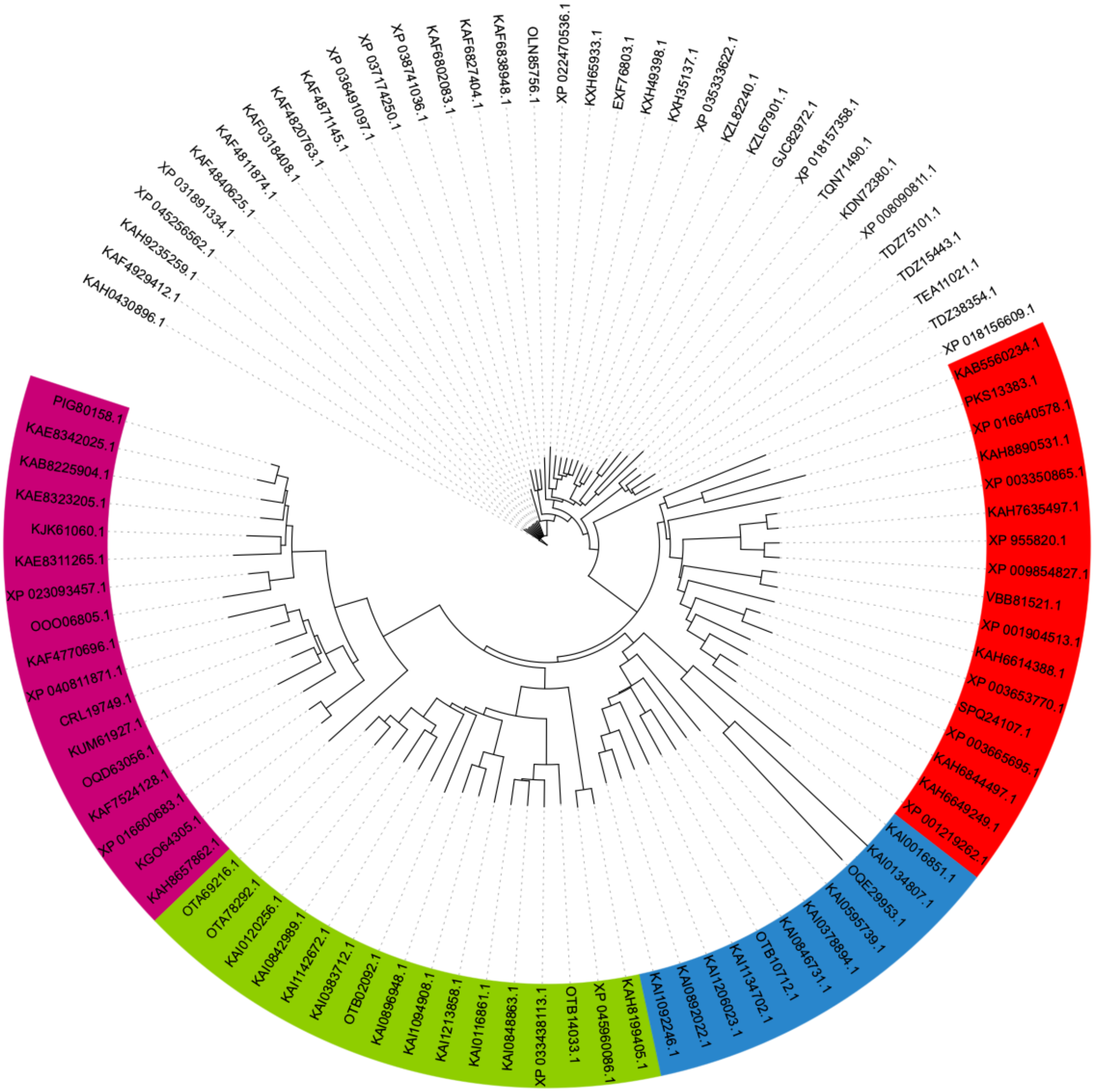

Figure S10. Neighbor joining tree of carboxylate reductases. Four different clades can be identified, and the respective sequence names are highlighted in purple, green, blue and red, respectively. Grouping of non-highlighted sequences is out of the scope of this work.

ConSurf server cited AlphaFold and ConSurf: Identification of Functional Regions in Proteins by Surface-Mapping of Phylogenetic Information (Figure S11).

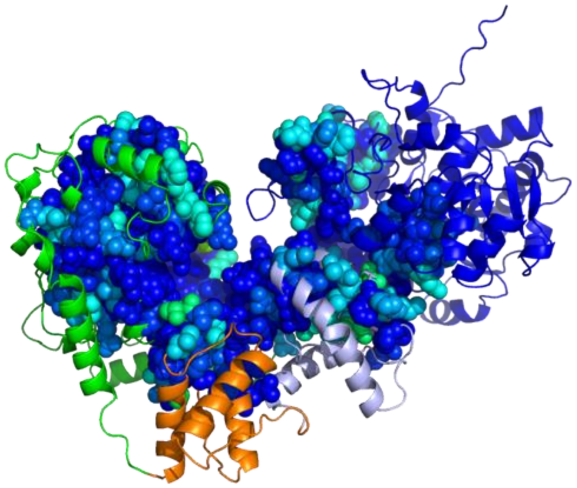

Figure S11. *Representation of highly conserved residues in the overall topology of full-length NcCAR. Residues that are conserved more than 90 % are depicted as spheres (color gradient: green 90 % conservation to blue 100 %). The coloring of the secondary elements is according to the respective subunits.*

9. References

(1) Schwendenwein, D.; Fiume, G.; Weber, H.; Rudroff, F.; Winkler, M. Selective Enzymatic Transformation to Aldehydes *in Vivo* by Fungal Carboxylate Reductase from *Neurospora crassa. Adv. Synth. Catal.* **2016**, *358* (21), 3414–3421. https://doi.org/10.1002/adsc.201600914.

(2) Stolterfoht, H.; Steinkellner, G.; Schwendenwein, D.; Pavkov-Keller, T.; Gruber, K.; Winkler, M. Identification of Key Residues for Enzymatic Carboxylate Reduction. *Front. Microbiol.* **2018**, *9*, 250. https://doi.org/10.3389/fmicb.2018.00250.

(3) Kabsch, W. XDS. *Acta Crystallogr. Sect. D Biol. Crystallogr.* **2010**, *66* (2), 125–132. https://doi.org/10.1107/S0907444909047337.

(4) Emsley, P.; Cowtan, K. Coot: Model-Building Tools for Molecular Graphics. *Acta Crystallogr. Sect. D Biol. Crystallogr.* **2004**, *60* (12 I), 2126–2132. https://doi.org/10.1107/S0907444904019158.

(5) Keegan, R. M.; Winn, M. D. MrBUMP: An Automated Pipeline for Molecular Replacement. *Acta Crystallogr. D. Biol. Crystallogr.* **2008**, *64* (Pt 1), 119–124. https://doi.org/10.1107/S0907444907037195.

(6) Hübschle, C. B.; Sheldrick, G. M.; Dittrich, B. ShelXle: A Qt Graphical User Interface for SHELXL. *J. Appl. Crystallogr.* **2011**, *44* (6), 1281–1284. https://doi.org/10.1107/S0021889811043202/TEXIMAGES/KK5092FI5.SVG.

(7) Cowtan, K. The Buccaneer Software for Automated Model Building. 1. Tracing Protein Chains. *urn:issn:0907-4449* **2006**, *62* (9), 1002–1011. https://doi.org/10.1107/S0907444906022116.

(8) Murshudov, G. N.; Skubák, P.; Lebedev, A. A.; Pannu, N. S.; Steiner, R. A.; Nicholls, R. A.; Winn, M. D.; Long, F.; Vagin, A. A. REFMAC5 for the Refinement of Macromolecular Crystal Structures. *Acta Crystallogr. Sect. D Biol. Crystallogr.* **2011**, *67* (4), 355–367. https://doi.org/10.1107/S0907444911001314.

(9) Manalastas-Cantos, K.; Konarev, P. V.; Hajizadeh, N. R.; Kikhney, A. G.; Petoukhov, M. V.; Molodenskiy, D. S.; Panjkovich, A.; Mertens, H. D. T.; Gruzinov, A.; Borges, C.; Jeffries, C. M.; Sverguna, D. I.; Frankea, D. ATSAS 3.0: Expanded Functionality and New Tools for Small-Angle Scattering Data Analysis. *J. Appl. Crystallogr.* **2021**, *54* (Pt 1), 343–355. https://doi.org/10.1107/S1600576720013412.

(10) Franke, D.; Petoukhov, M. V.; Konarev, P. V.; Panjkovich, A.; Tuukkanen, A.; Mertens, H. D. T.; Kikhney, A. G.; Hajizadeh, N. R.; Franklin, J. M.; Jeffries, C. M.; Svergun, D. I. ATSAS 2.8: A Comprehensive Data Analysis Suite for Small-Angle Scattering from Macromolecular

Solutions. *J. Appl. Crystallogr.* **2017**, *50* (Pt 4), 1212–1225. https://doi.org/10.1107/S1600576717007786.

(11) Konarev, P. V.; Volkov, V. V.; Sokolova, A. V.; Koch, M. H. J.; Svergun, D. I. PRIMUS: A Windows PC-Based System for Small-Angle Scattering Data Analysis. *J. Appl. Crystallogr.* **2003**, *36* (5), 1277–1282. https://doi.org/10.1107/S0021889803012779.

(12) Xie, X.; Watanabe, K.; Wojcicki, W. A.; Wang, C. C. C.; Tang, Y. Biosynthesis of Lovastatin Analogs with a Broadly Specific Acyltransferase. *Chem. Biol.* **2006**, *13* (11), 1161–1169. https://doi.org/10.1016/J.CHEMBIOL.2006.09.008.

(13) Younes, S. H. H.; Ni, Y.; Schmidt, S.; Kroutil, W.; Hollmann, F. Alcohol Dehydrogenases Catalyze the Reduction of Thioesters. *ChemCatChem* **2017**, *9* (8), 1389–1392. https://doi.org/10.1002/CCTC.201700165.

(14) Strohmeier, G. A.; Eiteljörg, I. C.; Schwarz, A.; Winkler, M. Enzymatic One-Step Reduction of Carboxylates to Aldehydes with Cell-Free Regeneration of ATP and NADPH. *Chem. – A Eur. J.* **2019**, *25* (24), 6119–6123. https://doi.org/10.1002/chem.201901147.

(15) Schwendenwein, D.; Fiume, G.; Weber, H.; Rudroff, F.; Winkler, M. Selective Enzymatic Transformation to Aldehydes *in Vivo* by Fungal Carboxylate Reductase from *Neurospora Crassa*. *Adv. Synth. Catal.* **2016**, *358* (21), 3414–3421. https://doi.org/10.1002/adsc.201600914.
